# The Association between Osteoarthritis and Occupational Clusters in the Korean Population: A Nationwide Study

**DOI:** 10.1371/journal.pone.0170229

**Published:** 2017-01-18

**Authors:** Hongdeok Seok, Sung Jae Choi, Jin-Ha Yoon, Gwan Gyu Song, Jong-Uk Won, Jae-Hoon Kim, Jaehoon Roh, Jae Hyun Jung

**Affiliations:** 1 Graduate School of Public Health, Yonsei University, Seoul, Korea; 2 Institute for Occupational Health, Yonsei University College of Medicine, Seoul, Korea; 3 Department of Preventive Medicine, Yonsei University College of Medicine, Seoul, Korea; 4 Korea University College of Medicine, Seoul, Korea; 5 Division of Rheumatology, Department of Internal Medicine, Korea University Ansan Hospital, Ansan, Korea; 6 Division of Rheumatology, Department of Internal Medicine, Korea University Guro Hospital, Seoul, Korea; University of Umeå, SWEDEN

## Abstract

Osteoarthritis (OA) is a considerable health problem worldwide. It is known to be associated with certain occupational risk factors. We examined the prevalence rate of OA by occupational cluster. Data were collected from the Korea National Health and Nutrition Examination Surveys (2010–2013). The total number of unweighted sample size was 9,905 participants: 4,460 men and 5,445 women, and OA prevalence was 5.3% and 18.4% respectively. OA patients were defined as participants with knee/hip joint pain and radiographic change of knee/hip joint. Occupational type was classified as either white, pink, blue, or green collar based on the occupational characteristics following physical demand: white for manager and professionals; pink for clerks and service/sales workers; blue for craft/trade workers, machine operators and assemblers, and elementary manual workers; and green for agricultural/fishery workers. We calculated the odds ratios (ORs) and 95% confidence intervals (95% CI) for the odds of a participant’s having OA according to the occupational cluster, with gender stratification. The multiple logistic regression model showed that, compared to the white collar group, the ORs of the pink, blue, and green collar workers were 1.23 (95% CI 0.64–2.36), 1.85 (95% CI 1.18–2.88), and 2.91 (95% CI 1.86–4.54), respectively, in males, and 2.53 (95% CI 1.71–3.73), 2.86 (95% CI 1.94–4.21), and 3.90 (95% CI 2.60–5.83), respectively in females. The prevalence rate of OA was associated with the occupational cluster, in order from highest to lowest: green, blue, pink, and white collar.

## Introduction

Osteoarthritis (OA) is a structural and functional degenerative joint disease involving the cartilage and the surrounding tissue [1]. OA is the most common joint disease in the world, with a prevalence of 10–15% of adults, and causes chronic pain and disability [2,3]. In a study in Korea, OA prevalence was 3.3% in males and 16.0% in females aged 50 years and older [4]. Because the average life span is gradually increasing, OA is also likely to increase with the size of the aged population. OA has considerable effects on social life and quality of life. OA has become an especially conspicuous problem in the recent era, in which people tend to actively work into old age.

Knees and hips are clinically frequent sites for developing OA [5,6]. Previous studies have shown that OA is associated with certain occupational risk factors, such as kneeling, squatting, climbing, heavy lifting, and vibration [7]. However, no study has examined whether OA is more or less likely to develop among people working in different occupational clusters. Generally, examination for OA has not been included in standard checkups, and OA has gone largely unobserved. Moreover, symptoms of OA are not correlated with radiologic findings [8,9], and severe radiological OA with pathological progression is often seen in patients who are asymptomatic or have mild symptoms.

OA is a slowly progressing disease, and the damaged cartilage is not able to recover normal status [5]; thus, prevention should be the focus. Early examination and diagnosis are necessary for the health of individuals and communities. Further, a finding that certain occupational clusters have higher prevalence rates of OA would indicate that greater awareness and caution regarding OA should be employed by these groups. We investigated OA prevalence by occupational cluster using data from the 2010–2013 Korean National Health and Nutrition Examination Survey (KNHANES).

## Materials and Methods

### Subjects

Data were collected from the Korea National Health and Nutrition Examination Survey (KNHANES), which collected data for 4 years between 2010 and 2013. Voluntary participants, who provided written informed consent, were involved in the KNHANES. The institutional review board of the Korea Centers for Disease Control and Prevention (KCDC) approved the study (IRB: 2010-02CON-21-C, 2011-02CON-06-C, 2012-01EXP-01-2C, and 2013-07CON-03-4C). The Korean Ministry of Health and Welfare conducted the KNHANES, which was a nationwide cross-sectional study. Households were randomly selected for participation, and sampled multi-stage stratification based on geographical areas. The KNHANES was conducted in accordance with the Helsinki Declaration of 2000.

In the 2010–2013 KNHANES, 11,969 participants were older than 50 and had received knee or hip joint X-rays; among these, we excluded soldiers, participants who did not have any economic activity during their lives, and those who did not complete the health survey section. Finally, in our study, unweighted sample size was 9,905 participants: 4,460 males and 5,445 females.

### Main variables

Knee/hip OA was defined based on knee/hip joint pain and radiographic changes of the knee/hip joints. Knee/hip joint pain was self-reported using the following question: “Have you experienced knee/hip pain for 30 days or longer over the past 3 months?” The radiographic criterion was a knee/hip joint Kellgren-Lawrence (KL) grade of 2 or higher [4].

Occupation was defined as the longest work in each participant’s life. Occupational type was classified as white collar for manager and professionals; pink collar for clerks and service and sales workers; blue collar for craft/trade workers, machine operators and assemblers, and elementary manual workers; and green collar for agricultural/fishery workers, based on the International Standard Classification of Occupations (ISCO) [10,11].

### Covariates

Sex, age, body mass index (BMI), alcohol consumption, smoking status, hypertension (HTN), diabetes mellitus, and income level were considered as potential confounding variables affecting OA [12, 13].

For obesity classification, according to the guidelines of KCDC, we classified the low-weight group for BMI lower than 18.5, the normal-weight group for BMI between 18.5 and 25, and the obese group for BMI higher than 25 [14]. Alcohol consumption status was defined as follows: heavy drinkers who consumed an average of 7 units and more of alcohol for men and 5 units and more for women 2 days and more per week, moderate drinkers who had consumed more than one glass of alcohol per month over the past year, and nondrinkers never drank or had drunk less than one glass of alcohol per month over the past year [15]. The smoking group comprised current smokers, and the nonsmoking group comprised those who had never smoked and former smokers. HTN was defined as average systolic blood pressure (SBP) 140 mmHg and over or diastolic blood pressure (DBP) 90 mmHg and over or taking antihypertensive medications, and pre-hypertension was defined as 120/80 mmHg and over. DM was defined as fasting plasma glucose (FPG) of 126 mg/dL, diagnosis as DM by a clinician, or taking an oral hypoglycemic agent or injected insulin. Impaired fasting glucose (IFG) was defined as not having DM and having FPG 100 mg/dL and more and 126 mg/dL or less. Income level was divided into quartile (lower, lower middle, upper middle and highest) based on questionnaire response.

### Statistical analysis

According to guidelines of the Korea Ministry of Health and Welfare and KCDC, we used survey weighted statistical analyses. We calculated the odds ratios (ORs) and 95% confidence intervals (95% CI) for OA according to occupation cluster with gender stratification. We used a multiple logistic regression model in which we controlled for the variables of age and BMI (Model I), and for additionally potential confounding variables of alcohol consumption, smoking status, BMI, HTN, DM, and income level (Model II). The *p*-values for trends about OA according to occupation in order white, pink, blue, green were also calculated using Cochran-Armitage trend test. The SAS 9.2 software (SAS Inc., Cary, NC, USA) was used for all statistical analyses, and a *p*-value of 0.05 or less was considered to indicate significance.

## Results

Table 1 provides the characteristic of study. The total number of weighted study subject were approximately 5.8million in males, approximately 6.1million in females. In males, 27.4% were white collar, 14.2% were pink collar, 41.3% were blue collar, and 17.1% were green collar. In females, 14.1% were white collar, 32.2% were pink collar, 34.2% were blue collar, and 19.5% were green collar. In males, 5.3% had OA, in spite of, in females, 18.4% had OA.

**Table 1:** Characteristic of study subject.

	Male			Female		
Variables	Frequency	Weighted frequency	%	Frequency	Weighted frequency	%
**Occupation**						
White	1,333	1,595,240	27.4	817	869,218	14.1
Pink	597	826,177	14.2	1,618	1,976,765	32.2
Blue	1,692	2,409,422	41.3	1,823	2,101,107	34.2
Green	838	994,424	17.1	1,187	1,196,959	19.5
**Age**						
50–59	1,675	3,062,052	52.6	2,270	3,029,426	49.3
60–69	1,569	1,690,270	29.0	1,756	1,707,930	27.8
70–79	1,090	953,179	16.4	1,214	1,170,240	19.0
80–99	126	119,763	2.0	205	236,452	3.9
**Obesity**						
Low	120	141,976	2.4	116	124,714	2.0
Normal	2,884	3,727,770	64.0	3,317	3,739,014	60.9
Obese	1,456	1,955,517	33.6	2,012	2,280,321	37.1
**Alcohol Intake**						
Nondrinking	1,418	1,689,947	29.0	4,000	4,386,662	71.4
Moderate drinking	2,398	3,125,848	53.7	1,370	1,650,726	26.9
Heavy drinking	644	1,009,469	17.3	75	106,660	1.7
**Smoking status**						
Never or past	3,043	3,749,803	64.4	5,240	5,862,932	95.4
Current	1,417	2,075,461	35.6	205	281,117	4.6
**Hypertension**						
Normal	1,017	1,384,927	23.8	1,505	1,839,241	29.9
Prehypertension	1,208	1,645,825	28.2	1,291	1,441,582	23.5
Hypertension	2,235	2,794,512	48.0	2,649	2,863,226	46.6
**Diabetes mellitus**						
Normal	2,229	2,912,358	50.0	3,429	3,836,833	62.4
Impaired fasting glucose	1,323	1,759,204	30.2	1,200	1,398,406	22.8
Diabetes mellitus	908	1,153,701	19.8	816	908,810	14.8
**Income**						
Highest	1,132	1,394,244	23.9	1,346	1,428,352	23.2
Upper middle	1,133	1,497,862	25.7	1,358	1,523,035	24.8
Lower middle	1,130	1,495,777	25.7	1,382	1,591,154	25.9
Lower	1,065	1,437,381	24.7	1,359	1,601,508	26.1
**Osteoarthritis**						
Yes	283	307,566	5.3	1,058	1,130,756	18.4
No	4,177	5,517,697	94.7	4,387	5,013,293	81.6
**Total**	4,460	5,825,263	100.0	5,445	6,144,049	100.0

Table 2 shows the demographics of unweighted study samples according to OA. In both male and female, occupation cluster, age, alcohol consumption, HTN, and income level had significant differences between OA group and non-OA group, while smoking status had no significant differences. BMI and DM had significant differences between OA group and non-OA group in female but not in male.

**Table 2:** Demographics of unweighted study samples according to OA. (n = 9905).

	Male (n = 4460)					Female (n = 5445)				
	OA (n = 283)		Non-OA (n = 4177)			OA (n = 1058)		Non-OA (n = 4387)		
	n	%	n	%	*p*-value	n	%	n	%	*p*-value
**Occupation**					< .001					< .001
White	45	3.38	1288	96.62		51	6.24	766	93.76	
Pink	24	4.02	573	95.98		274	16.93	1344	83.07	
Blue	113	6.68	1579	93.32		356	19.53	1467	80.47	
Green	101	12.05	737	87.95		377	31.76	810	68.24	
**Age**					< .001					< .001
50–59	39	2.33	1636	97.67		176	7.75	2094	92.25	
60–69	102	6.50	1467	93.50		372	21.18	1384	78.82	
70–79	118	10.83	972	89.17		422	34.76	792	65.24	
80–99	24	19.05	102	80.95		88	42.93	117	57.07	
**Obesity**					0.314					< .001
Low	7	5.83	113	94.17		18	15.52	98	84.48	
Normal	172	5.96	2712	94.04		502	15.13	2815	84.87	
Obese	104	7.14	1352	92.86		538	26.74	1474	73.26	
**Alcohol Intake**					0.001					< .001
Non-drinking	115	8.11	1303	91.89		827	20.68	3173	79.33	
Moderate drinking	123	5.13	2275	94.87		222	16.20	1148	83.80	
Heavy drinking	45	6.99	599	93.01		9	12.00	66	88.00	
**Smoking**					0.904					0.742
Never or Past	194	6.38	2849	93.62		1020	19.47	4220	80.53	
Current	89	6.28	1328	93.72		38	18.54	167	81.46	
**Hypertension**					0.021					< .001
Normal	48	4.72	969	95.28		179	11.89	1326	88.11	
Prehypertension	73	6.04	1135	93.96		210	16.27	1081	83.73	
Hypertension	162	7.25	2073	92.75		669	25.25	1980	74.75	
**Diabetes mellitus**					0.801					< .001
Normal	145	6.51	2084	93.49		606	17.67	2823	82.33	
Impaired fasting glucose	79	5.97	1244	94.03		214	17.83	986	82.17	
Diabetes mellitus	59	6.50	849	93.50		238	29.17	578	70.83	
**Income**					< .001					< .001
Highest	46	4.06	1086	95.94		224	16.64	1122	83.36	
Upper middle	67	5.91	1066	94.09		239	17.60	1119	82.40	
Lower middle	84	7.43	1046	92.57		274	19.83	1108	80.17	
Lower	86	8.08	979	91.92		321	23.62	1038	76.38	

**Fig 1:**
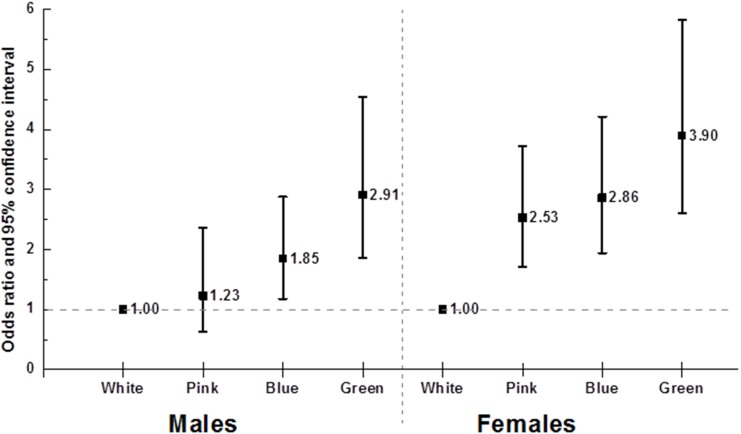
Osteoarthritis according to occupation clusters.

Compared to the white collar group, the ORs of the pink, blue, and green collar groups on OA were 1.27 (95% CI 0.67–2.38), 1.93 (95% CI 1.27–2.94), and 3.86 (95% CI 2.51–5.94), respectively, in males, and 3.41 (95% CI 2.32–5.02), 4.27 (95% CI 2.93–6.23), and 8.17 (95% CI 5.58–11.95), respectively, in females. In the multiple logistic regression model, adjusted for age and BMI (Model I) the ORs for pink, blue, and green collar were 1.34 (95% CI 0.71–2.53), 2.07 (95% CI 1.36–3.15), and 3.14 (95% CI 2.05–4.82), respectively, in males, and 2.66 (95% CI 1.81–3.93), 3.05 (95% CI 2.08–4.47), and 4.12 (95% CI 2.77–6.13), respectively, in females. In the multiple logistic regression model, adjusted for age, alcohol intake, smoking, BMI, hypertension, DM, and income level (Model II) the ORs for pink, blue, and green collar were 1.23 (95% CI 0.64–2.36), 1.85 (95% CI 1.18–2.88), and 2.91 (95% CI 1.86–4.54), respectively, in males, and 2.53 (95% CI 1.71–3.73), 2.86 (95% CI 1.94–4.21), and 3.90 (95% CI 2.60–5.83), respectively, in females. The *p*-value for the Cochran-Armitage trend test about OA according to occupation in order white, pink, blue, green was 0.001 or less in both males and females. (Table 3, Fig 1)

**Table 3:** Odds ratios and 95% confidence intervals of occupation on osteoarthritis among males and females.

	Males						Females					
	Crude		Model I ^a^		Model II ^b^		Crude		Model I ^a^		Model II ^b^	
Variables	OR	95% CI	OR	95% CI	OR	95% CI	OR	95% CI	OR	95% CI	OR	95% CI
**Occupation** ^**c**^												
White	1.00		1.00		1.00		1.00		1.00		1.00	
Pink	1.27	0.67−2.38	1.34	0.71 ~ 2.53	1.23	0.64 ~ 2.36	3.41	2.32−5.02	2.66	1.81 ~ 3.93	2.53	1.71 ~ 3.73
Blue	1.93	1.27−2.94	2.07	1.36 ~ 3.15	1.85	1.18 ~ 2.88	4.27	2.93−6.23	3.05	2.08 ~ 4.47	2.86	1.94 ~ 4.21
Green	3.86	2.51−5.94	3.14	2.05 ~ 4.82	2.91	1.86 ~ 4.54	8.17	5.58−11.95	4.12	2.77 ~ 6.13	3.90	2.60 ~ 5.83
**Age**												
50–59	1.00		1.00		1.00		1.00		1.00		1.00	
60–69	3.10	2.02 ~ 4.76	3.00	1.95 ~ 4.60	3.02	1.93 ~ 4.71	3.19	2.53 ~ 4.02	2.73	2.16 ~ 3.46	2.60	2.04 ~ 3.31
70–79	4.92	3.21 ~ 7.54	4.58	3.00 ~ 6.99	4.59	2.89 ~ 7.27	6.32	5.02~7.95	5.29	4.15 ~ 6.75	4.88	3.77 ~ 6.32
80–99	7.76	4.11 ~ 14.66	7.41	3.85 ~ 14.27	7.34	3.74 ~ 14.39	7.92	5.45~11.51	6.94	4.64 ~ 10.40	6.35	4.18 ~ 9.63
**Obesity**												
Low	1.00		1.00		1.00		1.00		1.00		1.00	
Normal	0.79	0.33~1.87	1.22	0.50 ~ 2.97	1.25	0.50 ~ 3.12	0.75	0.41 ~ 1.40	1.35	0.73 ~ 2.52	1.32	0.71 ~ 2.45
Obese	0.94	0.39 ~ 2.25	1.75	0.70 ~ 4.34	1.77	0.69 ~ 4.54	1.65	0.89 ~ 3.07	3.14	1.68 ~ 5.86	2.92	1.56 ~ 5.49
**Alcohol Intake**												
Nondrinking	1.00				1.00		1.00				1.00	
Moderate drinking	0.57	0.42 ~ 0.78			0.69	0.49 ~ 0.97	0.69	0.56 ~ 0.84			0.95	0.77 ~ 1.17
Heavy drinking	0.73	0.47 ~ 1.12			1.06	0.65 ~ 1.72	0.56	0.24 ~ 1.33			1.03	0.43 ~ 2.45
**Smoking**												
Never or past	1.00				1.00		1.00				1.00	
Current	0.99	0.73 ~ 1.35			1.26	0.90 ~ 1.75	0.89	0.58 ~ 1.38			1.10	0.68 ~ 1.78
**Hypertension**												
Normal	1.00				1.00		1.00				1.00	
Prehypertension	1.10	0.71 ~ 1.73			1.16	0.74 ~ 1.83	1.38	1.06 ~ 1.78			1.04	0.79 ~ 1.36
Hypertension	1.62	1.10–2.39			1.47	0.98 ~ 2.20	2.57	2.07 ~ 3.18			1.32	1.04 ~ 1.68
**Diabetes mellitus**												
Normal	1.00				1.00		1.00				1.00	
Impaired fasting glucose	0.90	0.64 ~ 1.28			0.95	0.66 ~ 1.37	1.03	0.83 ~ 1.26			0.78	0.62−0.98
Diabetes mellitus	0.85	0.59 ~ 1.23			0.78	0.54 ~ 1.14	1.96	1.59 ~ 2.42			1.13	0.89−1.44
**Income**												
Highest	1.00				1.00		1.00				1.00	
Upper middle	1.40	0.87 ~ 2.25			1.23	0.75 ~ 2.03	1.02	0.80 ~ 1.30			0.98	0.75 ~ 1.27
Lower middle	1.58	0.99 ~ 2.50			1.28	0.78 ~ 2.09	1.12	0.88 ~ 1.42			1.03	0.80 ~ 1.33
Lower	1.82	1.16 ~ 2.84			1.50	0.92 ~2.43	1.40	1.11 ~ 1.76			1.27	0.99 ~ 1.64

[OR = odds ratio; 95% CI = 95% confidence interval].

^**a**^ Adjusted for age and body mass index.

^**b**^ Adjusted for age, alcohol intake, smoking, body mass index, hypertension, diabetes mellitus, and income level.

^**c**^ P for trend of OA incidence according to occupation cluster < 0.001 in both male and female crude, model I, and model II.

## Discussion

We classified the occupations into four occupation-cluster groups by physical loading. The amount of manual labor, in order from highest to lowest, was green, blue, pink, and white collar group. Actually, our study showed that the green-collar group showed the highest prevalence rate of OA, followed by the blue-collar group, and then the pink-collar group. The white-collar group showed the lowest prevalence in both male and female. In the Cochran-Armitage trend test, about OA according to occupation in order white, pink, blue, and green, also statistically significant. On the basis of these results, we argued that physical labor can induce and develop OA. Particularly in the blue and green-collar groups, occupation was more strongly associated with OA than any other risk factor with the exception of age, including alcohol consumption, smoking status, obesity, hypertension, and DM (Table 2). Thus, occupation type was the second mostly important risk factor among the covariates tested in our study.

The results showed no differences in OA prevalence between white collar and pink-collar workers in males, but did see a difference in females. These results suggest that males in the pink collar group worked at similar levels to males in the white collar group, whereas females in the pink collar group had more physical activity, similar to females in the blue collar group.

Occupational classification in ISCO-08 was based on 10 major groups. Major Group 1: managers; Major Group 2: professionals; Major Group 3: technicians and associate professionals; Major Group 4: clerks; Major Group 5: service and sales workers; Major Group 6: skilled agricultural and fishery workers; Major Group 7: craft and related trade workers; Major Group 8: plant and machine operators and assemblers; Major Group 9: elementary occupations; and Major Group 0: armed forces occupations [16]. Occupation was re-classified into four groups according to the social and culture environment of Korea and skill level. White collar workers included Major Group 1, 2, and 3, pink included Major Group 4 and 5, green included Major Group 6, and blue included Major Group 7, 8, and 9 [17]. In Korea, green collar workers have much more physical activities than blue.

Females showed a higher prevalence rate of OA than males in this study. Compare to white collar group, odds ratios of pink, blue and green collar groups in females were 1.5−2 times higher than those in males. Srikanth et al.’s meta-analysis of gender differences in prevalence of OA similarly showed that females had a significantly higher incidence of knee and hip OA than males [18]. This finding suggests that hormonal factors may affect the development and progression of OA, and structural differences, such as knee–ankle angle and cartilage volume, may also influence OA [19,20]. Women have higher femoral lateral bowing in knee OA and increased rates of cartilage loss and progression of cartilage defects at the knee than men.

Additionally, in the current study, age was associated with OA prevalence in both males and females. Age and obesity are well known risk factors of OA [21,22]. OA is a degenerative disease where chondrocytes can perform only a limited number of replications. Because chondrocytes are the main factor associated with the development and progression of OA, OA is an inevitable consequence of aging [23]. A previous study using data from KNHANES 2010–2012 found that smoking was a risk factor of OA in Korean males [4]. However, we include data of KNHANES 2013 and found that smoking was not associated with OA. Furthermore, inverse correlation was reported between smoking and knee OA [24,25]. Bae et al. showed that HTN was also associated with OA using KNHANES 2007–2009 data [26]. However, the findings of these earlier studies using KNHANES 2007–2009 data were not consistent with those of the current study.

Alcohol eventually has a pathological effect on joints and increases OA development; both animal and case-control studies have verified this relationship between alcohol consumption and OA [27,28]. However, previous study showed that alcohol consumption was not associated with OA. DM was not associated with OA in either gender in this study. Overweight status has been shown to be strongly associated with OA, and physical activity tends to lower BMI [29,30]. Therefore, the fact that the green and blue collar occupations involved higher levels of physical activity may have influenced the results of this study.

Our study has some strengths. First, this study was based on authoritative nationwide data (KNHANES 2010−2013). Thus, our study can show recent Korean status and tendencies of relationship between OA and occupational clusters. In addition, we defined OA as a combination of clinical symptoms and radiologic findings. Radiologic OA considers only pathophysiological joint signs present in radiographic images, whereas symptomatic OA considers joint pain or crepitus [31,32]. These two components are both important in the diagnosis of OA. We also examined the association of OA with occupation type according to gender stratification. As mentioned earlier, gender is an important factor of OA development and progression. Finally, this study establishes relationship between occupational clusters and the prevalence of OA, which was the first study, to the best of our knowledge.

Some limitations are present in this study. First, the cross-sectional design precludes conclusions about causal relationships, so further prospective studies and intervention trials should be undertaken to establish a causal association between occupational cluster and OA prevalence. However, the relationship of OA to occupation is important in that the progression of OA makes physical activity difficult, limiting OA patients’ ability to engage in occupation-related physical activities. Second, the occupational clusters were based on self-reports. Consequently, these data may have been influenced by systematic errors in individuals’ consideration of their occupations, which may have led to non-differential misclassification or underestimation. However, the questionnaire subdivided occupation into four groups and presented examples of each occupational cluster, fostering participants’ selections of the correct occupational types.

The present study found that the prevalence rate of OA is associated with occupational cluster. The prevalence of OA, in order from highest to lowest, was observed in the green, blue, pink, and white collar occupation clusters. Therefore, workers in occupational clusters at high-risk for OA should use caution and preventative measures for OA. Additionally, early diagnosis and prevention systems for OA should be prepared to improve public health care.
